# Comparative outcomes of oophoropexy techniques in preventing recurrent adnexal torsion

**DOI:** 10.1007/s00404-025-08128-x

**Published:** 2025-07-28

**Authors:** Yael Yagur, Amit Klein, Lisa Tiosano, Omer Weitzner, Yair Daykan, David Rosen, Zvi Klein, Ron Schonman

**Affiliations:** 1 Gynecology Division, Department of Obstetrics and Gynecology, Meir Medical Center, 59 Tchernichovsky St., Kfar Saba, Israel; 2 School of Medicine, Faculty of Medical and Health Sciences, Tel Aviv University, Tel Aviv, Israel; 3 Sydney Women’s Endosurgery Centre (SWEC), St George Hospital, Kogarah, Sydney, Australia; 4 University of New South Wales, Sydney, Australia

**Keywords:** Adnexal torsion, Oophoropexy, Recurrent torsion, Ovario-round ligament fixation

## Abstract

**Purpose:**

To compare the effectiveness of ovarian ligament plication and ovario-round ligament fixation ("hot-dog" technique) in preventing recurrent adnexal torsion.

**Methods:**

This is a retrospective cohort study performed in the gynecology department, of a university-affiliated tertiary medical center. Women of reproductive age who underwent first fixation following adnexal torsion surgery on the ipsilateral side between 2014 and 2023. The study included a homogenous population with normal ovarian size. Primary outcome was the recurrence rate of adnexal torsion following each fixation technique. Two fixation techniques of ovarian ligament plication and ovario-round ligament fixation were compared.

**Results:**

A total of 525 adnexal torsion events were reviewed, involving 413 patients. After applying strict inclusion criteria, 38 patients who underwent their first fixation on the ipsilateral side were included in the final analysis. Among them, 27 (71%) underwent ovarian ligament plication, and 11 (29%) underwent ovario-round ligament fixation. Recurrent torsion occurred in 25.9% (7 of 27) of patients in the ovarian ligament plication group, while no recurrences were observed in the ovario-round ligament fixation group. While clinically promising, the difference did not reach statistical significance (*p* = 0.08). Fixation was also safely performed during pregnancy, primarily in the first trimester, with no postoperative complications observed.

**Conclusions:**

Ovario-round ligament fixation shows promise as a technique for preventing recurrent torsion with a favorable safety profile. Due to the limited sample size, further larger-scale studies are required to confirm these findings and comprehensively evaluate long-term outcomes.

## What does this study add to the clinical work

**Table:** 

Ovario-round ligament fixation was associated with no recurrences in recurrent adnexal torsion, compared with a 25.9% recurrence rate after ovarian ligament plication. It demonstrated a safety profile even when performed during early pregnancy. Further studies needed for validation.	

## Introduction

Adnexal torsion is a gynecological emergency with an estimated prevalence of 2–6% and is frequently observed in women of reproductive age [1–3]. Urgent surgical intervention is the recommended course of action for patients suspected of having adnexal torsion to preserve ovarian function and future fertility, and prevent complications [4]. The surgical procedure typically involves adnexal detorsion. Additional procedures that may be performed include cystectomy, salpingectomy, or oophorectomy depending upon the cause of the torsion. The final treatment decision is based on such factors as the patient's age, family planning considerations, and surgical findings [5, 6].

Despite the gravity of the initial episode, recurrent adnexal torsion looms as a distinct concern, with rates varying from 15% in the general population to as high as 70% among premenarchal and adolescent individuals following conservative management [7–10]. While risk factors for the initial occurrence of adnexal torsion have been extensively studied, factors contributing to its recurrence are less well understood. Several such factors have been identified for primary instances of ovarian torsion, including age, presence of ovarian cysts or masses, enlarged ovaries (due to hyperstimulation or polycystic ovary syndrome), differences in size between the ovaries, and pregnancy [1, 11]. A history of torsion with normal adnexa is a known risk factor for contralateral torsion [12], while there appears to be an inverse relationship between recurrent torsion and age [13].

To prevent the recurrence of adnexal torsion, several techniques for oophoropexy have evolved. While no clear guidelines exist for ovarian fixation, the best known is ovarian fixation, where the ligament is plicated and shortened [14]. Other fixation methods described are to the pelvic sidewall or the uterosacral ligaments [15, 16] with a significant failure rate reported in some studies [17, 18]. A relatively novel approach, ovario-round ligament fixation, involves fixation of the ovarian to the round ligament with a non-absorbable suture. No large series have been reported to evaluate the efficacy of this technique nor the preferred suture material to be employed [19, 20].

Given the limited guidelines and lack of consensus on optimal methods for managing recurrent adnexal torsion, this study aims to assess the effectiveness of different fixation techniques in preventing recurrence.

## Materials and methods

### Study population

This retrospective cohort study compared the rate of adnexal torsion reoccurrence after one of two ovarian fixation techniques performed in a 10-year period (2014–2023) in a single tertiary referral center.

All included study participants underwent emergency laparoscopic surgery for suspected ovarian or tubal torsion. The study included all cases with confirmed surgical adnexal torsion during reproductive years and fixation following a previous torsion. The surgical types of fixation included ovarian ligament plication and ovario-round ligament fixation (“hot-dog”) techniques. Only patients presenting with physiologic and transient cysts, specifically corpus luteum, follicular, and hemorrhagic corpus luteum cysts, were included. In our clinical protocol, cysts deemed pathological were treated with cystectomy rather than oophoropexy fixation. This approach was designed to maintain a homogeneous study population. Patients with non-confirmed torsion during surgery or torsion involving the contralateral adnexa were excluded. In addition, patients who underwent a second or subsequent oophoropexy (i.e., repeat fixation procedures) were excluded, as the study focused solely on the first fixation performed following recurrent torsion.

Follow-up duration ranged from 1 to 10 years and was based on clinical documentation available through hospital electronic medical records. Recurrence events were identified through these records. While follow-up duration varied between patients, all included cases had at least 12 months of follow-up to minimize underreporting of recurrence.

The study compared recurrent torsion rates between patients who underwent ovarian ligament plication and those who underwent ovario-round ligament fixation.

### Outcomes

The primary outcome was to compare ovarian ligament plication and ovario-round ligament fixation techniques, focusing on the recurrence rate. The secondary outcome aimed to examine differences in patient characteristics, surgical details, and additional interventions between the groups.

### Study protocol

Patients who presented to the gynecological emergency room were selected for the study based on symptoms, physical examination, and sonographic evaluation suggestive of adnexal torsion. All participants included in the study underwent emergency laparoscopic surgery with diagnosed torsion.

If there was a history of prior torsion on the same side and no clear reason for torsion such as an ovarian cyst that could be treated with cystectomy or oophorectomy (based on the patient’s age), the patient underwent oophoropexy as part of the procedure.

Two primary oophoropexy techniques with non-absorbable sutures were used: (1) ovarian ligament plication: this technique involved shortening of the ovarian ligament; (2) ovario-round ligament fixation: this technique involved fixation of the utero-ovarian ligament to the round ligament.

Non-absorbable sutures: ethibond, prolene, and silk were employed to provide a permanent solution to prevent future recurrence.

### Surgical techniques

All surgeries were performed via laparoscopy under general anesthesia. Following identification and detorsion of the twisted adnexa, the decision to perform oophoropexy was made in cases of recurrent torsion with no clear underlying pathology requiring resection. Two fixation techniques were used.

In the ovarian ligament plication technique, the utero-ovarian ligament was folded onto itself and shortened using a non-absorbable suture placed in an interrupted or figure-of-eight fashion to reduce ligament length and limit ovarian mobility.

In the ovario-round ligament fixation technique, a non-absorbable suture was used to approximate the utero-ovarian ligament to the ipsilateral round ligament. A double stitch was placed between the two structures. This technique is also referred to as the "hot-dog in a bun" configuration, due to the alignment of the ovary between the two ligaments.

All procedures were performed by senior gynecologic surgeons with expertise in minimally invasive surgery.

### Data

The dataset included demographic, clinical, sonographic, and surgical information obtained from electronic medical records. The parameters included were age, smoking status, obstetrics history (gravidity, parity, abortion status, history of cesarean section), clinical parameters (nausea, vomiting, bowel symptoms, the onset of acute abdominal pain), vaginal examination data, sonographic parameters (ovarian size and variability between them, edema, whirlpool, Doppler flow, and ovarian cysts), laboratory parameters [white blood cells (WBC) (K/µL), hemoglobin level (g/dL), and C-reactive protein (CRP) (mg/L)], and data on the side of torsion and number of rotations during surgery, type of suture, and recurrence rate were documented.

### Ethics

This study was conducted according to good clinical practice guidelines and was approved by the Meir Medical Centre Institutional Review Board. Due to the retrospective nature of the study, waiver for informed consent was accepted based in the nature of the study.

### Statistical analysis

Patient characteristics were compared between the groups. All statistical analyses were performed using R software. Categorical variables are presented as numbers and percentages, while continuous variables are reported as medians with interquartile ranges or means with standard deviations, as appropriate. Categorical variables were compared using the Chi-square test or Fisher’s exact test, while continuous and ordinal variables were analyzed using the independent samples *t* test or Mann–Whitney *U* test.

Due to the small sample size, especially in the ovario-round ligament fixation group, we were unable to perform multivariate regression analysis to control for potential confounding variables.

All statistical tests were two-sided, and a *p* value of < 0.05 was considered statistically significant.

All statistical analyses were conducted using R version 4.2.2., an open-source programming language for statistical computing maintained by the R Core Team and supported by the R Foundation for Statistical Computing (Vienna, Austria).

## Results

**Fig. 1:**
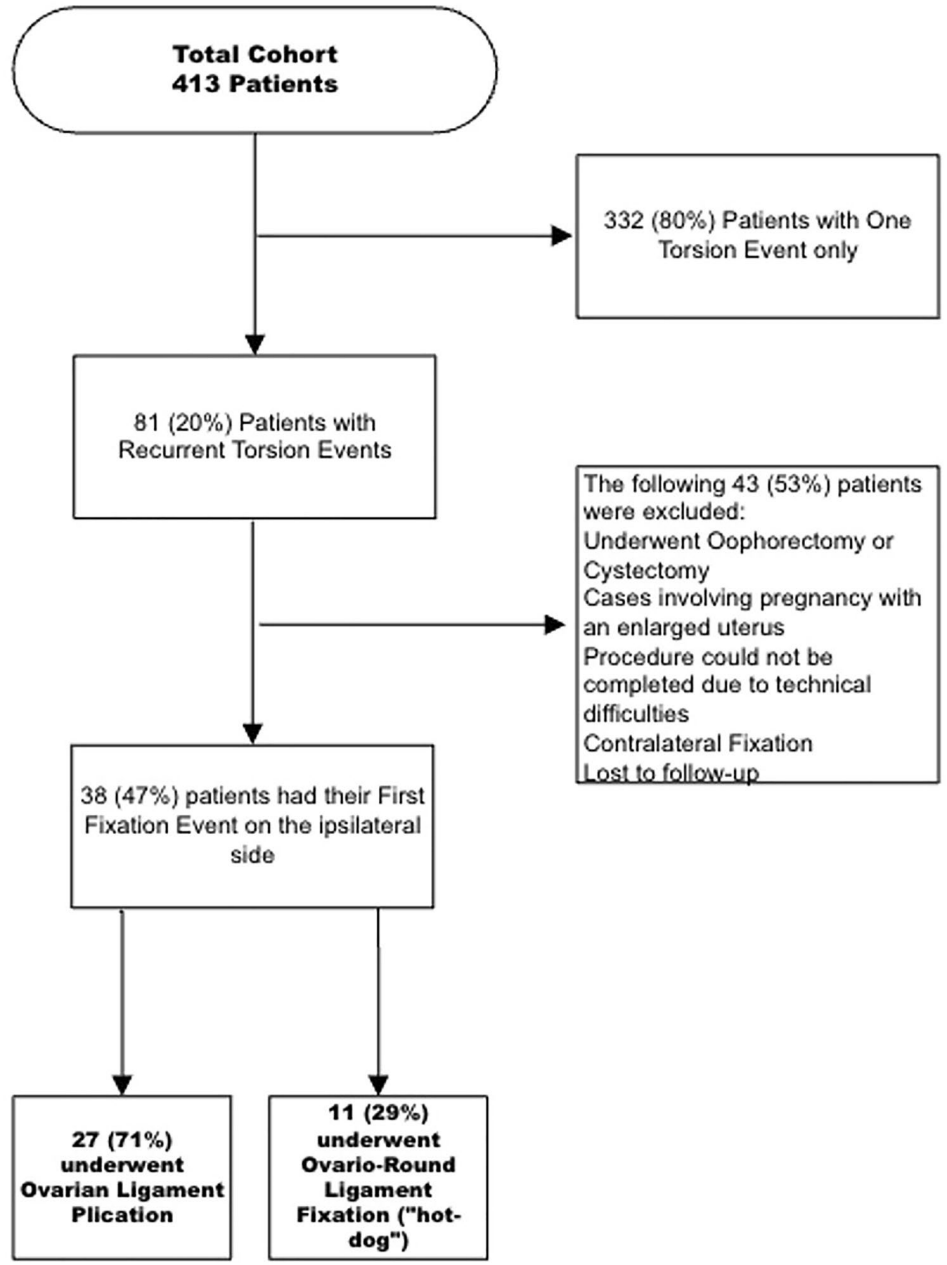
Study population

During the study period, a total of 413 patients, and 525 events of adnexal torsion were included in the cohort. Of these, 332 (80%) experienced a primary torsion event, while 81 (20%) had recurrent torsion events. Of the 81 patients with recurrent torsion, 43 (53%) were excluded for the following reasons: undergoing oophorectomy or cystectomy, pregnancy with an enlarged uterus, technical difficulties preventing completion of the procedure, contralateral fixation, or loss to follow-up. The remaining 38 patients (47%) underwent their first fixation event on the affected side. Among these, 27 patients (71%) underwent ovarian ligament plication, while 11 patients (29%) underwent ovario-round ligament fixation (the "hot-dog in a bun" technique) (see Fig. 1). Of the total cohort (525 events), 64 (12.2%) oophoropexy events for repeated torsion.

Table 1 presents the demographic characteristics of the study groups. There were no significant differences between the ovarian ligament plication and ovario-round ligament fixation groups in terms of age, smoking status, BMI, family status, primiparity, history of previous cesarean delivery, or use of contraception. Both groups included patients with normal menstrual cycles and those who were pregnant at the time of diagnosis, 7 of 8 in the first trimester with no complication during the same pregnancy.

**Table 1:** Demographic characteristics of the study groups

Characteristic	Ovarian ligament plication (*n* = 27)	Ovario-round ligament fixation (n = 11)	*p *value
Age (years)	28 [22.5, 31.0]	33 [24.0, 33.5]	0.23
Smoker	2 (13.3%)	1 (14.3%)	1
Family status			
Single	9 (45%)	4 (40%)	1
Married	15 (55.6%)	6 (60%)	
BMI (kg/m^2^)	19.2 [18.6, 21.6]	21.3 [20.75, 21.85]	0.36
Primiparity	14 (56%)	4 (40%)	0.53
History of previous CD	2 (8%)	1 (10%)	0.65
Normal cycles	14 (58.3%)	6 (75%)	0.84
Contraception			
IUD	4 (15.4%)	1 (9.1%)	
OCP	3 (11.5%)	0 (0%)	
Condom	1 (3.8%)	1 (9.1%)	0.7
None	18 (69.2%)	9 (81.8%)	
Pregnancy at diagnosis^a^	7 (25.9%)	1 (9,1%)	0.39

Data are shown as number (%), mean ± standard deviation or median [range], as appropriate

*BMI* Body mass index, *CD* Cesarean delivery, *OCP* Oral contraception

^a^ Six of seven in first trimester, one in second trimester (non utero-ovarian ligament plication group), and one in first trimester (utero-ovarian ligament plication group)

Table 2 presents the patient characteristics at diagnosis in those cases in which fixation had been performed. The most common symptom was sudden abdominal pain. Vomiting, nausea, and bowel symptoms were also reported but showed no significant differences between the groups. On physical examination, finding of localized abdominal pain was very common (85–100%) and adnexal tenderness was observed in up to 50% of the cases.

**Table 2:** Patient characteristics at oophoropexy surgery

Characteristic	Ovarian ligament plication (*n* = 27)	Ovario-round ligament fixation (n = 11)	*p *value
Symptoms prior to previous surgery^a^			
Sudden abdominal pain	12 (92.3%)	8 (100%)	1
Vomiting	8 (29.6%)	7 (63.6%)	0.07
Nausea	12 (44.4%)	6 (54.5%)	0.72
Bowel symptoms	2 (7.4%)	2 (18.2%)	0.56
Physical examination findings prior to previous surgery			
Peritonitis	8 (30.8%)	4 (36.4%)	1
Localized abdominal pain	23 (85.2%)	11 (100%)	0.67
Adnexal tenderness	13 (50%)	3 (27.3%)	0.13
Uterine tenderness	3 (11.1%)	0 (0%)	0.2
Heart rate at diagnosis	90 [84.5, 98.0]	92 [76.5, 95.5]	0.41
Blood test results at diagnosis			
WBC (K/µL)	9.3 [8.1, 10.5]	10.1 [8.1, 11.7]	0.38
Hemoglobin (g/dL)	11.2 [10.7, 11.8]	12.2 [11.8, 12.4]	0.002
CRP (mg/L)	1.1 [1.0, 1.7]	0.9 [0.1, 1.1]	0.18
US Imaging prior to surgery			
Uterine size	62 [57.0, 78.0]	76.0 [67.5, 77.0]	0.5
Right ovary measurement	45 [34.0, 67.0]	44 [32.0, 62.0]	0.38
Left ovary measurement	37 [32.0, 50.5]	40 [25.0, 51.5]	0.81
Edema	19 (79.2%)	8 (80%)	1
Ovarian cyst^b^	11 (42.3%)	8 (72.7%)	0.15
Cyst size (mm)	27 [21.5, 40.3]	30 [24.5, 35.5]	0.6
Whirlpool sign	11 (73.3%)	5 (55.6%)	0.41
Doppler flow	12 (70.6%)	6 (75%)	1

Data are shown as number (%), mean ± standard deviation or median [range], as appropriate

*WBC* White blood cells; US ultrasound, *CRP* C-reactive protein

^a^All symptoms started between 1 and 2 h prior to presentation

^b^Ovarian cyst types: corpus luteum, follicular, and hemorrhagic corpus luteum cysts

Blood tests at diagnosis, including WBC (K/µL) and CRP levels (mg/L), showed no significant differences between the groups. Hemoglobin level (g/dL) was significantly higher in the ovario-round ligament fixation group. Ultrasound imaging revealed similar findings. Overall, ovarian measurements on both sides were up to 45 mm. Ovarian functional cyst types included corpus luteum, follicular cysts and hemorrhagic corpus luteum. The whirlpool sign, an indicator of torsion, was observed in up to 73.3% of cases with no significant difference. Ovarian edema was observed in 80% of cases.

Table 3 presents the surgical characteristics and outcomes of patients undergoing oophoropexy. No significant differences were observed in the side of torsion (right vs. left) or tubal involvement. The time interval between the detorsion surgery (without fixation) and the current oophoropexy was similar across groups. Suture types used for fixation included Ethibond, Prolene, and Silk, with Ethibond being the most commonly used in both groups with no significant differences. Subsequent torsion events following oophoropexy were observed only in the ovarian ligament plication group, while no recurrences were reported in the ovario-round ligament fixation group, this finding did not reach the level of statistically significance (25.9% vs 0%, respectively; *p* = 0.08).

**Table 3:** Data form oophoropexy surgery

Characteristic	Ovarian ligament plication (*n* = 27)	Ovario-round ligament fixation (*n* = 11)	*p *value
Number of torsion*s* prior to fixation			
1	24 (88.9%)	10 (90.9%)	1
> 1 (2 or 3)	3 (11.1%)	1 (9.1%)	
Torsion side			
Right	17 (63%)	8 (72.7%)	0.71
Left	10 (37%)	3 (27.3%)	
Tubal involvement	9 (33.3%)	3 (27.3%)	1
Number of rotations			
1	5 (18.5%)	3 (27.3%)	0.11
2	13 (48.1%)	6 (54.4%)	
3	7 (25.9%)	2 (18.2%)	
4	2 (7.4%)	0 (0%)	
Time from previous surgery to oophoropexy surgery (months)	7.00 [0.75, 21.00]	8.5 [3.0, 72.0]	0.29
Side of oophoropexy			
Bilateral	1 (4.8%)	1 (10%)	1
Right	7 (33.3%)	3 (30%)	
Left	13 (61.9%)	6 (60%)	
Other interventions were performed			
Ovarian cystectomy	5 (21.7%)	1 (14.3%)	1
Suture type			
Ethibond	19 (76%)	8 (80%)	0.53
Prolene	4 (16%)	2 (20%)	
Silk	2 (8%)	0 (0%)	
Additional torsion events following oophoropexy	7 (25.9%)	0 (0%)	0.08

Data are shown as number (%), mean ± standard deviation or median [range], as appropriate

## Discussion

Adnexal torsion is a gynecological emergency with a risk of recurrence and complications, particularly in young women and those with normal adnexa [4, 21]. Despite its clinical importance, there is no consensus on the most effective surgical technique to prevent recurrence [8, 13, 22]. Our study directly addresses this gap by comparing two fixation methods ovarian ligament plication and ovario-round ligament fixation in a homogeneous cohort of women of reproductive age, all of whom underwent their first fixation for torsion on the ipsilateral side with normal-sized ovaries. No recurrences were observed in the ovario-round ligament fixation group, compared to a recurrence rate of 25.9% in the ovarian ligament plication group. Although clinically meaningful, this difference did not achieve statistical significance.

The general recurrence rate for adnexal torsion ranges from 19 to 38%, which is consistent with our observed overall recurrence rate [23]. The literature shows higher recurrence rates in populations with normal ovarian size and morphology, particularly in premenarchal and adolescent girls, likely due to ligamentous laxity and smaller adnexal structures [7, 10]. Additionally, women undergoing assisted reproductive techniques and pregnant women are at higher risk due to ovarian hyperstimulation and enlarged ovaries [22].

While many studies have explored the recurrence rates of adnexal torsion in general and the accompanying risk factors, the recurrence rate following oophoropexy is a less extensively studied area. Our study specifically examined a homogeneous population of patients who underwent their first oophoropexy on the ipsilateral side of a previous torsion, with normal adnexa. We observed a recurrence rate of 18.4% across all fixation types. Smorgick et al. reported a high recurrence rate of 78.9% in patients with otherwise normal adnexa after utero-ovarian ligament shortening, primarily in premenarchal and adolescent populations, though based on a much smaller sample size and more specific population [17]. Another study reported a recurrence rate of 29.6% following oophoropexy across all fixation types in a similarly small cohort, but unlike our study, it did not exclusively include first fixation cases or torsion on the ipsilateral side [13]. The strength of our study lies in its relatively larger, well-defined cohort and its focused inclusion criteria.

The most significant finding of our study is the absence of recurrence in the ovario-round ligament fixation group, compared to a recurrence rate of 25.9% in the ovarian ligament plication group. Although this difference did not reach statistical significance, it suggests a trend favoring the ovario-round ligament fixation technique. The ovario-round ligament fixation method is considered less disruptive to normal ovarian anatomy compared to other fixation methods [23] and has been proposed as a strategy to reduce recurrence. Obut et al. described this approach involving utero-ovarian ligament folding and fixation to the round ligament, which was successful in his report [19]. However, most available evidence on this technique comes from small case reports and limited series, offering low levels of evidence. One study reported no statistically significant difference between fixation methods [13] despite a higher failure rate observed with ovarian ligament plication compared to ovario-round ligament fixation (35% vs. 20%). Our study contributes meaningful data by including a relatively larger and well-defined cohort of patients who underwent first-time fixation on the ipsilateral side with normal adnexa. This focused design strengthens the observed trend and supports the potential advantage of the ovario-round ligament technique. These findings provide an important basis for further research and support the consideration of this technique in future clinical guidelines.

In addition, we also examined the potential impact of suture material on the success by evaluating different non-absorbable suture types used during the procedure, a factor that has not been addressed in the previous literature. Our findings suggest that the type of suture material does not significantly influence the stability or long-term success of the fixation technique.

Our study also focused on clinical and sonographic factors associated with adnexal torsion, complementing the analysis of surgical parameters. Fixation during pregnancy poses unique challenges [15], as the altered anatomy and hormonal changes can potentially increase the risk of complications. However, in our study, fixation performed during pregnancy was shown to be feasible, particularly during the first trimester, with no postoperative complications observed in this subgroup for either technique.

Our study shows that the most frequent clinical finding is localized pain in one quadrant upon abdominal palpation. Notably, adnexal torsion is less likely to present with signs of peritonitis, emphasizing the importance of a comprehensive assessment. The diagnostic process requires an understanding of the complete clinical picture, with a major emphasis on sonographic evaluation [24, 25]. We emphasized the whirlpool sign, absence of Doppler flow, and ovarian edema aiding diagnosis. These findings are consistent with the current published literature [24].

The findings of this study have several implications for clinical practice and future research. While our findings suggest that ovario-round ligament fixation may reduce recurrence rates, concerns regarding long-term ovarian function remain underexplored. Further studies are needed to evaluate functional outcomes, including chronic pelvic pain, fertility potential, and the risk of adhesions, following different fixation strategies. Prospective, multicenter research with long-term follow-up would help guide best practices in managing recurrent adnexal torsion.

A strength of our study is the homogeneous nature of the population. It included women of reproductive age who underwent their first fixation for torsion on the ipsilateral side, with normal-sized ovaries. Our dataset represents one of the largest homogeneous cohorts to date focusing on first-time fixation with normal adnexa. Additionally, our study demonstrates that no cases of recurrent torsion were observed following ovario-round ligament fixation, suggesting this technique’s potential as a more effective approach for recurrence prevention, even though statistical significance was not achieved, likely due to the small sample size. Another strength lies in the detailed assessment of clinical and sonographic data. Furthermore, our findings reinforce the feasibility and safety of fixation during pregnancy.

Despite its strengths, our study has limitations. The retrospective nature of our study may limit the ability to control for confounding variables. We acknowledge that the small sample size of the ovario-round ligament fixation group limits the statistical power of our study and the strength of the conclusions. This limitation restricts our ability to detect statistically significant differences and prevents the use of multivariate regression analyses to control for potential confounders, which constitutes a vulnerability in assessing causality. Consequently, our findings should be regarded as preliminary and hypothesis-generating. Although the study demonstrates a clinically meaningful trend favoring the ovario-round ligament fixation technique, caution is warranted in interpreting these results as definitive evidence of superiority. Larger, multicenter prospective studies are essential to validate these observations and to guide clinical practice. Additionally, questions remain regarding the long-term safety of this treatment approach. Specifically, the potential impact of fixation on ovarian reserve and the functionality of the fallopian tubes remains unclear. Long follow-up periods to assess the effects of adnexal fixation on reproductive health are needed. Furthermore, our study did not evaluate long-term complications such as chronic pelvic pain, dyspareunia, or ovarian function. Although no pain-related symptoms were documented during follow-up, this remains an important limitation. We recommend that future prospective studies include thorough assessment of pain. Another limitation is that all procedures in our center are performed by a senior expert in minimally invasive gynecologic surgery. However, in cases where the procedure is performed by a trainee without direct supervision by a senior specialist, there is a potential risk of ureteral injury, which should be carefully considered. Finally, although a comparison to non-fixation might be theoretically informative, it is not clinically acceptable in current practice, as fixation is considered standard of care for recurrent torsion in our institution. Therefore, our study design comparing two fixation methods reflects real-world management and provides relevant clinical insight.

In conclusion, our study suggests the potential benefits of ovario-round ligament fixation in preventing recurrent torsion, with no cases of recurrence observed in this group. However, due to the limited sample size and retrospective design, these findings should be interpreted with caution. Additionally, the technique appears to be safe when performed during pregnancy, with no postoperative complications observed. Further large-scale studies are needed to validate these results and address the long-term safety and fully assess reproductive implications of adnexal fixation techniques.

## Data Availability

The authors confirm data supporting the findings of this study are available within the manuscript. Additional supporting data are available from the corresponding author upon reasonable request.
